# An Editorial on Revolutionizing Urinary Care in Pakistan by Embracing the United Kingdom National Health Service Approach to Catheterisation Documentation and Trial Without Catheter

**DOI:** 10.7759/cureus.73243

**Published:** 2024-11-07

**Authors:** Syed Haseeb Ullah Jan Bacha, Saman Babar, Muhammad Hamza Khan, Shawal Hameed

**Affiliations:** 1 Medicine, East Sussex Healthcare National Health Service Trust, East Sussex, GBR; 2 Physiology, Abbottabad International Medical Institute, Abbottabad, PAK; 3 Medicine, Lady Reading Hospital Medical Teaching Institution, Peshawar, PAK

**Keywords:** acute urinary retention (aur), catherization, : clinical audit, editorial, medical documentation, nhs guidelines, pakistan, trial without catheter, uk - united kingdom, urinary catheter

## Abstract

This editorial highlights the importance of catheterisation documentation and the practice of trials without catheters in improving patient outcomes in Pakistan. Acute urinary retention, a major urological emergency, needs short-term catheter placement, with a trial without a catheter used globally to reduce catheter-associated urinary tract infections. This procedure, often enhanced by alpha-1 blockers, enables patients to regain continence and promotes micturition post-catheterisation, particularly useful following surgeries such as prostatectomy. However, trials without catheters remain underutilised in Pakistan, particularly outside urology departments, potentially leading to severe complications such as acute renal failure and urosepsis. Accurate documentation of catheterisation details, including catheter size, insertion time, clinical indications, and any complications, is paramount for minimising risks and improving continuity of care. A clinical audit led by Dr. Syed Haseeb Ullah Jan Bacha at Eastbourne District General Hospital highlighted critical gaps in catheterisation documentation, revealing lapses in recording crucial parameters like bladder scan volumes and staff names. Recommendations from the audit, including educational interventions and system improvements, necessitate the need for precise record-keeping, a practice standard in the United Kingdom but lacking in Pakistan. Introducing trial without catheter (TWOC) and documentation practices can elevate patient safety and foster a culture of continuous improvement in Pakistan’s healthcare system.

## Editorial

Introduction

We are writing this editorial to highlight the importance of effectively conveying the significance of catheterisation documentation and the practice of trials without catheters (TWOC) to healthcare practitioners in Pakistan.

TWOC

Acute urinary retention (AUR) is one of the top three urological emergencies, and it often requires the placement of a urinary catheter for short-term use [1]. To avoid catheter-associated urinary tract infections (CAUTIs), clinicians use the TWOC strategy [2,3]. TWOC is a standard urological procedure practised worldwide, allowing the patient to voluntarily empty the bladder after removing the catheter. The addition of an alpha-1 blocker doubles the chances of a successful TWOC [4,5]. This strategy allows patients to regain continence or use alternative continence aids, such as pads or penile sheaths. It is also used to check if urinary output is enough in admitted patients after surgeries, such as prostatectomy. It can also be performed at the patient's request [4]. Please note, do not proceed with TWOC if hourly measurement of urine output is required, pre- or post-operatively, in case of suprapubic catheter and end-of-life [6].

TWOC technique

After a TWOC procedure, patients are asked to urinate twice within six hours, and their urine output is measured to ensure bladder function. Proper technique is crucial for both men and women to ensure complete bladder emptying. For proper bladder emptying in females, they are asked to be relaxed and sit with their feet flat on the floor while their elbows remain on their thighs. They are instructed to avoid hovering over the toilet and count to 10, lean forward slightly, and try to pee again, or stand up, move around, and sit down to pee again. This double voiding technique helps ensure the female’s bladder is completely emptied. Standing with feet slightly apart and staying relaxed is best for male patients. After micturition, they should count to 10, lean forward, or apply gentle pressure to the lower abdominal region to ensure the bladder is fully emptied. Intermittent catheter clamping is an alternative practice in some medical centres. Still, studies have not shown it to be more successful than TWOC, and it is also associated with complications such as urinary tract injury and increased duration of catheter placement [7,8].

Lack of TWOC practice in Pakistan

Our main aim is to raise awareness among healthcare providers in Pakistan that TWOC is not routinely practised in most hospitals, especially outside of the Urology Departments. Although it may be used in nephrology units, its implementation remains limited. Failing to ensure that patients can urinate before discharge can lead to serious complications, such as acute renal failure and urosepsis [9].

Catheterisation documentation (reference from National Health Service (NHS) clinical audit) 

Precise catheterisation documentation is essential for improving patient health, as it captures details such as the type and size of the catheter, date and time of insertion, clinical rationale, and any observed complications. This record helps reduce the risk of infection and other complications by ensuring all healthcare providers clearly understand the procedure specifics, supporting accurate care. Furthermore, this transparency enhances continuity of care, enabling hospitals to evaluate outcomes and refine practices to provide safer, more effective patient experiences.

**Figure 1:**
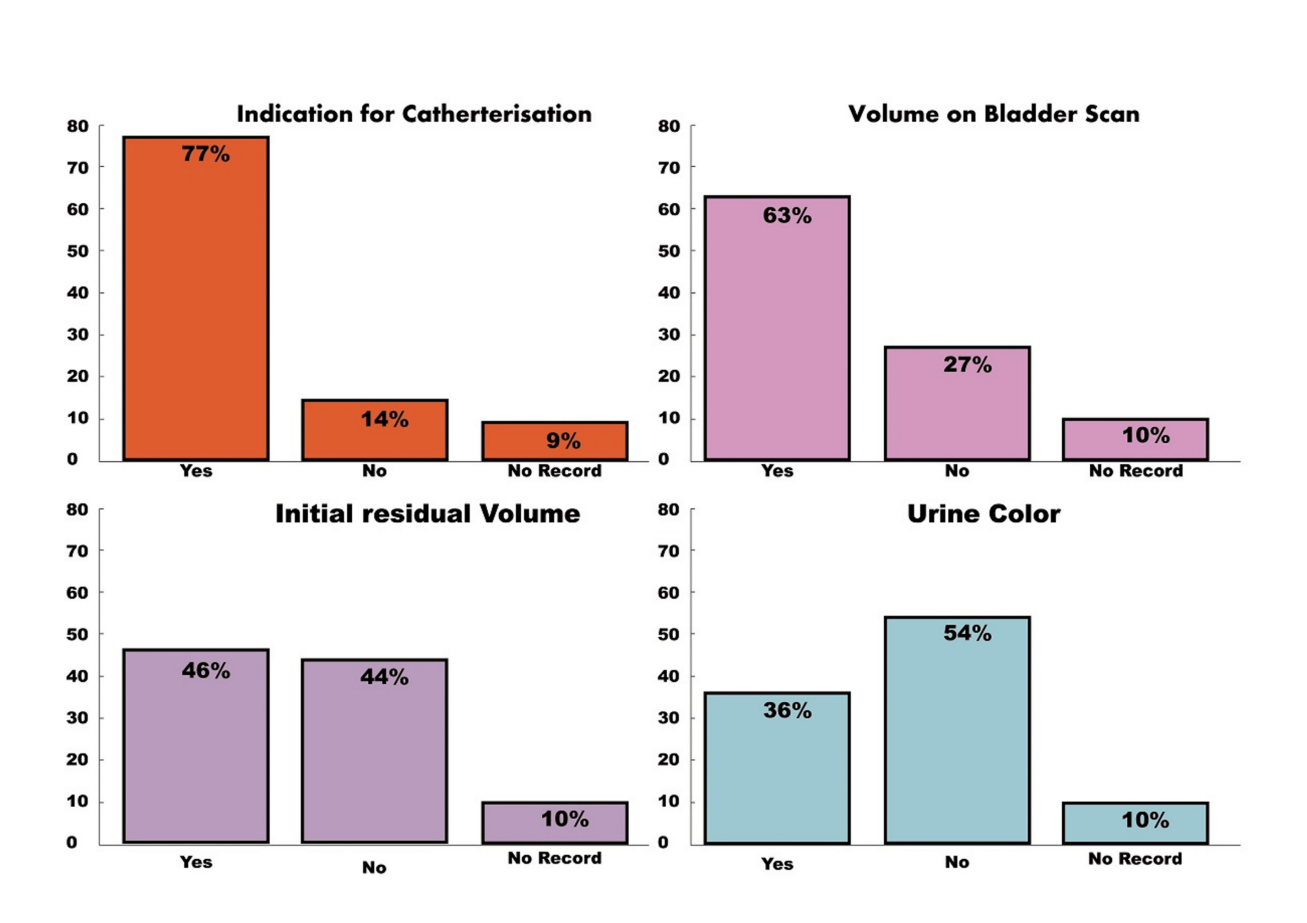
Percentage of indication for catheterisation, bladder scan residual volume, initial residual volume, and urine colour documented and not documented by hospital staff at Eastbourne General Hospital, National Health Services

**Figure 2:**
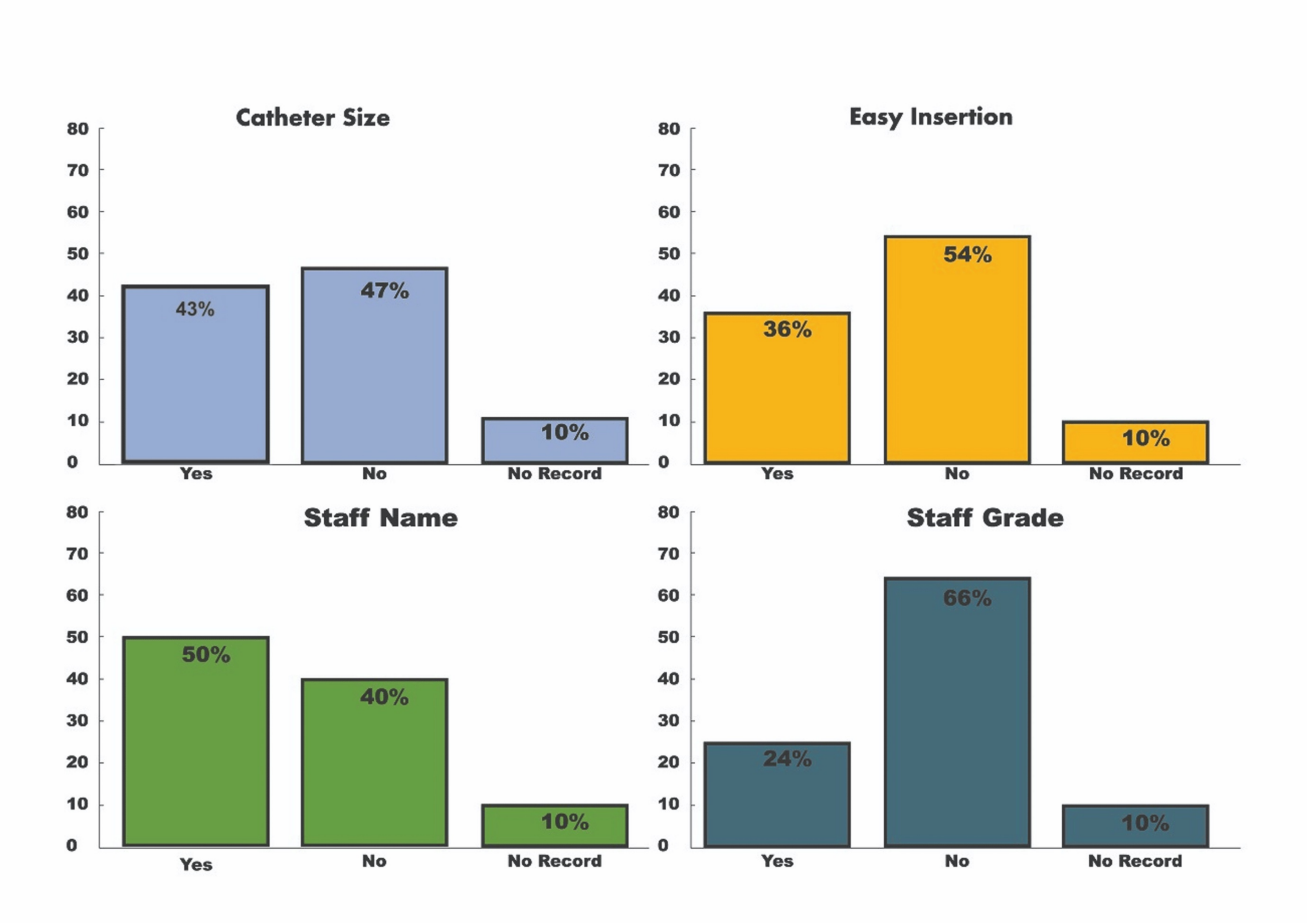
Percentage of catheter size, ease of insertion, staff name, and staff grade documented and not documented by hospital staff at Eastbourne General Hospital, National Health Service

To reinforce this point, we refer to the clinical audit titled *'Assessing the Adherence to Documentation Standards for Patients Catheterised in the Emergency Department and Wards at Eastbourne District General Hospital'*. This audit was conducted by Dr Syed Haseeb Ullah Jan Bacha, the first author of the editorial, and his Urology team [10]. The first cycle of the audit found several shortcomings in catheterisation documentation. The indication for catheterisation was documented in 77% of cases, but 9% had no record. Documentation of bladder scan volume was inconsistent, with 63% completed by staff and 10% left unrecorded. Only 46% of hospital staff documented initial residual volume, while 44% was filled in by others, and 10% remained missing. The documentation of urine colour was even lower, with only 36% completed by staff, and catheter size and ease of insertion were properly recorded in only 43% and 36% of cases, respectively. Staff names were recorded in 50% of cases, while in 10% of cases, staff names were missing, and staff grades were recorded in only 24% of cases, as shown in Table 1 and Figures 1-2.

**Table 1:** Name and percentage of indicators documented and not documented for urinary catheterisation by hospital staff at Eastbourne District General Hospital, National Health Service

		Percentage of indicators		
S. no.	Indicators	Filled by hospital staff	Not filled by hospital staff	No record
1.	Indication for catheterisation	77%	14%	9%
2.	Volume on bladder scan	63%	27%	10%
3.	Initial residual volume	46%	44%	10%
4.	Urine colour	36%	54%	10%
5.	Catheter size	43%	47%	10%
6.	Easy insertion	36%	54%	10%
7.	Staff name	50%	40%	10%
8.	Staff grade	24%	60%	10%

These findings highlighted major lapses in documentation, especially in terms of catheter size, insertion ease, and staff identification. Recommendations like involving educational interventions during staff induction and system enhancements in collaboration with the Nerve Centre team were proposed. This audit provided necessary evidence reflecting the need for proper documentation in catheterisation procedures, which aligns with established practices in the United Kingdom, where clinical audits are routinely done to address any gaps that arise in clinical practice. In contrast, Pakistan faces a critical lack of proper documentation and an established auditing culture.

Conclusion

TWOC and maintaining clear, complete records when placing catheters can improve patient care in Pakistan. TWOC help patients regain bladder control after catheter use, lowering the risk of infections. Good documentation, like that practised in the NHS, ensures that all steps and details are clear for healthcare providers, helping prevent mistakes and maintain good care. By bringing these practices into routine, Pakistan’s healthcare system can achieve higher standards, fostering a culture focused on patient safety and continuous improvement.
